# The effect of supporting carbons on the gas phase synthesis of octahedral Pt_3_Ni electrocatalysts with various H_2_:CO ratios

**DOI:** 10.1038/s41598-022-16742-x

**Published:** 2022-07-22

**Authors:** L. Payattikul, L. Intakhuen, T. Kiatsiriroat, K. Punyawudho

**Affiliations:** 1 Department of Mechanical Engineering, Faculty of Engineering, Chiang Mai University, Chiang Mai, 50200 Thailand; 2 Graduated School, Chiang Mai University, Chiang Mai, 50200 Thailand; 3 Energy Harvesting and Storage Laboratory, Mechanical Engineering, Chiang Mai University, Chiang Mai, 50200 Thailand; 4 Center of Clean Energy Development for Green, Faculty of Engineering, Chiang Mai University, Chiang Mai, 50200 Thailand

**Keywords:** Fuel cells, Materials for energy and catalysis, Nanoscale materials, Nanoscale materials

## Abstract

The gas phase synthesis of octahedral Pt_3_Ni/C electrocatalysts using several carbon substrates (Ketjen black, Graphene, and Vulcan XC-72R) was investigated. Different carbon substrates altered the morphology and alloy of Pt_3_Ni nanoparticles, with octahedral morphology and alloy metal preferentially developing on Ketjen black and Graphene, while spherical shape and bimetallic metal preferentially developing on Vulcan. Furthermore, the shape was shown to be regulated throughout the reduction process, with the H_2_:CO ratio playing a crucial role in controlling octahedral morphology and carrying out the ORR activity. At a 1:3 H_2_:CO ratio, the Pt_3_Ni/Ketjen black exhibited the highest ORR activity for both mass activity (1.02 A mgPt^−1^) and specific activity (5.09 mA cm^−2^) that were 16.5 and 66.1 times larger than commercial Pt/C catalysts, respectively (0.062 A mgPt^−1^ and 0.077 mA cm^−2^). The best ORR activity of Pt_3_Ni onto Graphene and Vulcan XC-72R was exhibited with a 1:1 H_2_:CO mixture. The catalysts were tested using a 4000-voltage-cycle accelerated durability test, and the Pt_3_Ni/Ketjen catalyst fared the best in terms of ORR stability and durability.

## Introduction

PEMFCs (polymer electrolyte membrane fuel cells) are a type of fuel cells that has received a lot of attention in recent years. Electrocatalysts, which assist the oxygen reduction reaction (ORR) and hydrogen oxidation reaction (HOR) at the cathode and anode, respectively, are a vital component of PEMFC^1–7^. Platinum nanoparticles are the most general electrocatalysts for PEMFCs because to their high catalytic activity^3–9^. The ORR for platinum nanoparticles is commonly referred to as a sluggish kinetic, and it is the most important hindrance to the energy conversion efficiency of state-of-the-art PEMFCs^10–15^. As a result, electrocatalytic research continues to focus on ORR enhancement.

It has been reported that the addition of other metals (M) onto Pt as Pt-M nanoparticles catalysts has been shown to boost ORR activity. Nickel (Ni) is one of these metals that is commonly employed because it is affordable, easy to get, and has good electrochemical properties^13,14,16–25^. Using density functional theory (DFT) calculations and surface-sensitive examination, Stamenkovic et al.^26^ theoretically reported that the Pt_3_Ni in single crystal surface with (111) facet has an excellently high ORR catalytic activities, which is 10 times higher than Pt (111) surface and 90 times higher than Pt/C commercial catalyst. Following that, several studies have been carried out and published on the synthesis process of platinum-nickel nanoparticles with (111) facet or octahedral shape (octahedral Pt_x_Ni/C), which demonstrate excellent ORR catalytic activity^10,13,17,19,27–41^. The synthesis process has been classified primarily into two categories: liquid phase synthesis (i.e., called solvothermal method)^10,31,32,34–41^ and gas phase synthesis (i.e. called solid-state chemistry)^27–29,33^.

Liquid-phase technique generally utilizes organic surfactants to regulate the octahedral shape. Organic surfactants commonly contaminate the catalyst surface, necessitating additional removal operations that drive up production costs and make this technique more complex and financially uncompetitive. Furthermore, liquid-phase synthesis has a poor scaling-up capacity, making it unsuitable for large-scale manufacture of the octahedral Pt_x_Ni catalyst. On the other hand, a scalable, less complicate, and low-cost technique for mass manufacture of octahedral Pt_x_Ni alloy nanoparticles has been established using gas-phase synthesis^27,33^. Furthermore, this technique makes it easier to manage the atomic composition of metals and is a good way to minimize metal particle aggregation^42^. Colloidal Pt_x_Ni nanocrystals have intrinsic limitations due to their ligand-covered surfaces, which not only restrict free access to surface active sites but also obstruct electron transport between the catalyst and support, lowering total ORR performance. Growing nanoparticles directly on carbon supports is an effective and straightforward synthetic way to overcome this problem^10,43^. Although carbon black such as Vulcan XC-72R has been commonly employed as a support, different carbon blacks/carbons with higher specific surface area (such as Ketjen black) and superior electrical conductivity (such as Graphene) may increase ORR electrocatalytic activity. These carbon supports will impact the octahedral formation and particle size related to the H_2_ and CO ratio due to their distinct surface characteristics, leading the homogeneity between Pt and Ni to become either alloy or bimetallic catalysts, which has never been reported previously.

In this work, we used a gas phase synthesis to create octahedral Pt_3_Ni nanoparticles supported on several carbon substrates (Ketjen black, Graphene, and Vulcan XC-72R). To regulate octahedral form and particle size, H2 and CO mixed gases with a Pt:Ni atomic ratio of 3:1 were examined. Transmission electron microscopy (TEM) was used to disclose the physical structure as well as the line scanning with energy dispersive X-ray (EDX) analysis. The X-Ray diffraction (XRD) analysis evaluated the alloy and bimetallic metal between Pt and Ni. In addition, the ORR activity related to specific activity (SA) and mass activity (MA) was estimated by electrochemical analysis. The accelerated durability test (ADT) was carried out in saturated oxygen up to 4000 cycles.

## Experimentals

### Preparation of octahedral Pt_3_Ni nanoparticles supported on carbon black

Pt and Ni precursors were impregnated carbon supports, and then the reduction was carried out in the gas phase, resulting in octahedral Pt_3_Ni catalysts on carbon. First, the metal precursors of platinum (II) acetylacetonate (Pt(acac)_2_, 48.0% of Pt, Alfa Aesar) and nickel (II) acetylacetonate (Ni(acac)_2_, 95%, Sigma-Aldrich) were dissolved in acetone (C_3_H_6_O, 99.5%, RCI Labscan) with molecular ratio of Pt:Ni at 3:1. Ketjen black (Cabot), Graphene (Cheap Tubes), and Vulcan XC-72R (Cabot) carbon supports were added to the solution and shaken for one hour. The acetone was then evaporated, leaving a dry combination of metals impregnated on carbons. For 1 h at 200 °C, the reduction was carried out in a combination of hydrogen (H_2_, 99.9%, Linde) and carbon monoxide (CO, 99.9%, Labgaz). By altering the gases volumetric flow rate, the effect of H_2_:CO composition on Pt_3_Ni nanoparticles was investigated. The overall flow rate of H_2_:CO was in the range of 100 to 140 cm^3^ min^−1^.

### Physical characterization

The specifics surface area of carbon supports was examined by the brunauer–emmett–teller (BET) analysis. The inductively coupled plasma-atomic emission spectrometry (ICP-OES, Perkin Elmer, Optima 7300DV) was used to measure the metal loadings of Pt and Ni. The morphologies and dispersion of Pt_3_Ni nanoparticles on various carbons were observed by transmission electron microscopy (TEM, JEOL JEM-2010 at 200 kV). The atomic percentage and the elemental distributions were characterized by energy dispersive spectroscopy which operated on TEM (TEM-EDS, JEOL JEM-2100Plus at 200 kV). The crystalline structure of prepared nanoparticles was analyzed using X-ray diffraction analysis (XRD, Rigaku, SmartLab).

### Electrochemical properties characterization

The electrochemical properties of prepared Pt_3_Ni/C electrocatalysts were evaluated by the voltammetry technique via a standard three-electrode system at room temperature using a bi-potentiostat (Pine Instrument Co.). A glassy carbon of the rotating disk (GC-RDE) was a working electrode (GC-RDE, Pine Research Co.), whereby a reference electrode was a single junction silver chloride electrode (Ag/AgCl, Pine Research Co.) in 3 M KCl. A counter electrode was a platinum wire located nearby the working electrode. The catalysts inks were prepared using 10 mg of Pt_3_Ni/C mixed in 5 ml of stock solutions, which properly composed of deionized water (RCI Labscan), isopropanol (C_3_H_8_O (IPA), RCI Labscan), and Nafion solution (5 wt% of nafion solution, 1000 EW, Dupont). The catalysts inks were sonicated for well dispersion for 20 min, and then, carefully dropped onto GC-RDE (i.e. working electrode), which had physical surface area of 0.19625 cm^2^. The Pt loading on GC-RDE were kept constant around 40 µg_Pt_ cm^−2^. The cyclic voltammetry (CV) was applied to evaluate the hydrogen under potential deposition (HUPD), which was a measurement of the electrochemical active surface areas (ECSA). The CVs were carried on under He-saturated in 0.1 M perchloric acid (HClO_4_, 70%, Loba Chemie Pvt. Ltd.) as the supporting electrolyte at a scanning rate of 50 mV s^−1^ at room temperature. The linear sweep voltammetry (LSV) technique was used for measuring the oxygen reduction reaction (ORR) of catalytic activities. The LSV was carried out under O_2_-saturated in 0.1 M HClO_4_ electrolyte at a scanning rate of 20 mV s^−1^ with a rotation speed of 1600 rpm. The electrode was activated by CV for 40 cycles prior to LSV. The mass activity (MA) and specific activity (SA) of electrocatalysts were calculated using kinetics current (i_k_). The polarizations of ORR were corrected with the iR_soln_ drop, and the background (b.g.) currents were also corrected according to the Ref.^9^. Furthermore, the ORR stability was carried on via the accelerated durability test (ADT) by running the CV and LSV up to 4000 cycles (about 22 h. for each sample). The CVs were operated in the voltage range from 0.4 to 1.2 V_SHE_ at a scanning rate of 50 mV s^−1^. The LSVs were operated at a scanning rate of 20 mV s^−1^ from 0.2 to 1.2 V_SHE_. In addition, the electrochemical properties of commercial Pt/C catalysts (20% of Pt on Vulcan XC-72, Premetek Co.) were compared with the same conditions.

## Results and discussions

### Preparation of octahedral Pt_3_Ni using H_2_:CO ratios and carbon supports

Ketjen black (K), Graphene (G), and Vulcan XC-72R (V) were the three types of supported carbons studied. According to BET study, Ketjen black has the largest specific surface area with 1499.11 m^2^ g^−1^, followed by Graphene and Vulcan XC-72R with 887.37 m^2^ g^−1^ and 259.08 m^2^ g^−1^, respectively. ICP was used to measure the mass loadings of Pt and Ni during impregnation (Table 1). For all carbons, Pt mass loading ranged from 18.25 to 19.29 wt%, whereas Ni mass loading ranged from 1.66 to 1.88 wt%. These weight ratios were around 3:1 for Pt and Ni, respectively, in terms of atomic loading ratios. It means that the Pt and Ni compositions were kept reasonably consistent and similar even if varied carbon was supported.

**Table 1:** Specifics surface area of carbon supports, metals loading and metals atomic percentage of the prepared Pt_3_Ni/C.

Catalysts	Specifics surface area of supported carbons (m^2^ g^−1^)	Metals loading (wt%)		Metals atomic percentage (at%)	
		Pt	Ni	Pt	Ni
Pt_3_Ni/Ketjen black	1499.11	18.62	1.88	76.19	23.81
Pt_3_Ni/Graphene	887.37	18.25	1.87	76.54	23.46
Pt_3_Ni/Vulcan XC-72R	259.08	19.29	1.66	78.33	21.67

**Figure 1:**
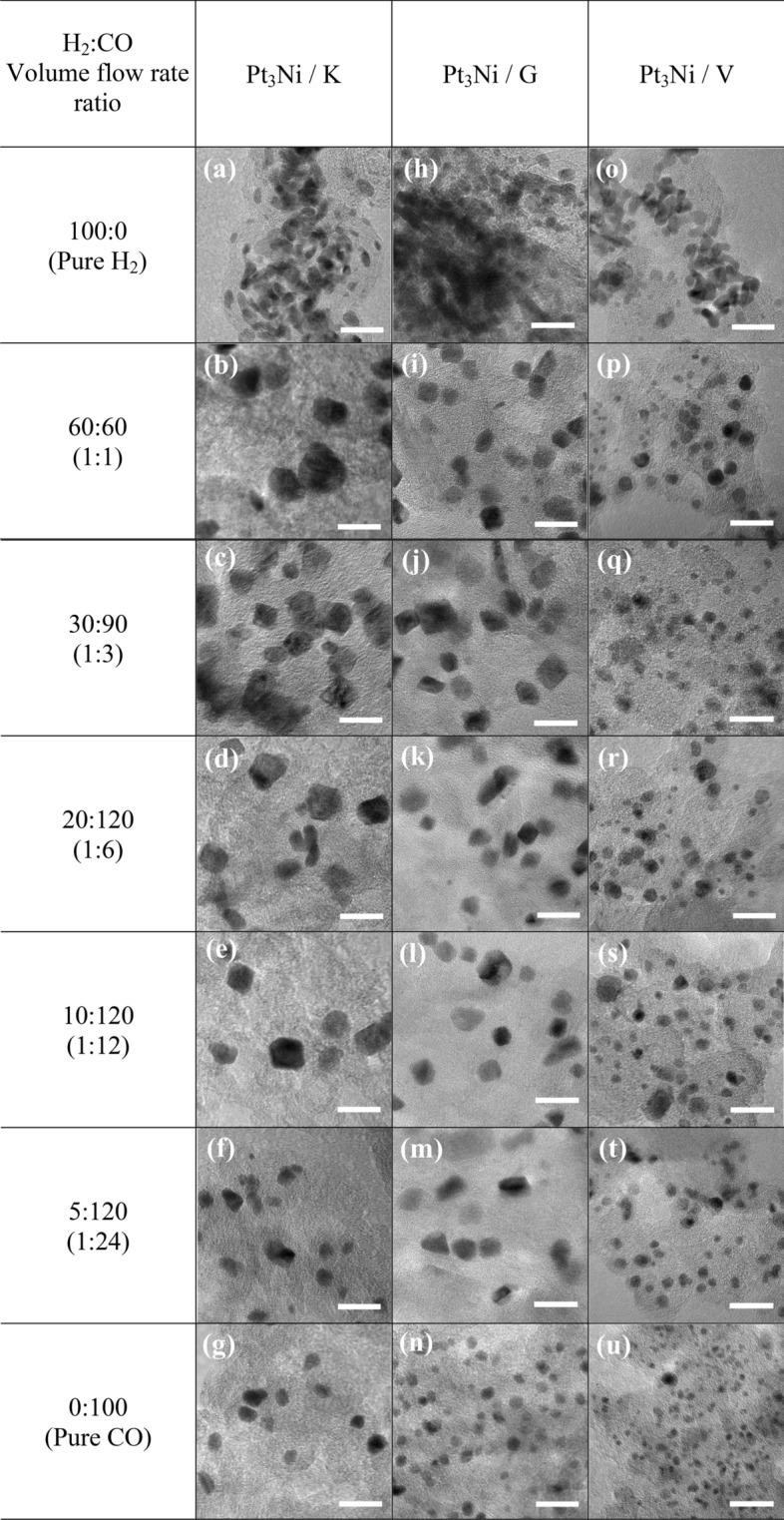
The TEM images of Pt_3_Ni/C nanoparticles reduced with different H_2_:CO volume flow rate (cm^3^ min^−1^) ratios; (**a**)–(**g**) for Ketjen black; (**h**)–(**n**) for Graphene; and (**o**)–(**u**) for Vulcan XC-72R carbon supports. The scale bar in all TEM images represents 20 nm.

**Figure 2:**
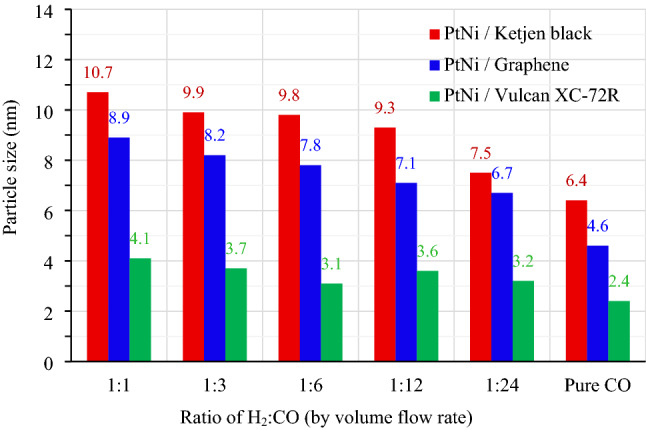
The average particle size of Pt_3_Ni/C nanoparticles prepared with different H_2_:CO volume flow rate (cm^3^ min^−1^) i.e., 60:60 (1:1), 30:90 (1:3), 20:120 (1:6), 10:120 (1:12), 5:120 (1:24) and pure CO, respectively.

All impregnated carbons were reduced in H_2_ and CO environments with volume flow rates (cm^3^ min^−1^) ranging from 100:0 (pure H_2_) to 60:60 (1:1), 30:90 (1:3), 20:120 (1:6), 10:120 (1:12), 5:120 (1:24) and 0:100 (pure CO). As shown in Fig. 1, the Pt_3_Ni nanoparticles with varying H_2_:CO ratios were photographed by TEM at the same magnification of 400,000 times. Figure 1a–g shows the morphology of Pt_3_Ni/K electrocatalysts, whereas Fig. 1h–n and Fig. 1o–u show the morphology of Pt_3_Ni/G and Pt_3_Ni/V electrocatalysts, respectively. For all supported carbons, when the reducing gas was pure H_2_, as shown in Fig. 1a,h,o, the particles of Pt_3_Ni congregated near to one other and probably agglomerated to form the lump of Pt metals, especially in Graphene support. As soon as the CO was mixed into the reducing gas, the Pt_3_Ni particles were well dispersed with various shapes and sizes at particular H_2_:CO ratio. Pt_3_Ni average particle sizes from TEM images on Ketjen black, Graphene, and Vulcan supports with varied H_2_:CO ratios are shown in Fig. 2. Pt_3_Ni particles on Ketjen black had the highest size, regardless of the H_2_:CO ratio, whereas those on Graphene and Vulcan were smaller and smaller, respectively. This might be related to the specific surface area of supports, which, as indicated in Table 1, is Ketjen > Graphene > Vulcan. However, this finding differed significantly from pure Pt and Pt-Ru particle size, which shrank as the specific surface area of supported carbon increased^44,45^. Furthermore, the results demonstrate that as CO content grew until pure CO was reached, Pt_3_Ni particle size in all supports did reduce.

The formation of Pt_3_Ni particles changed considerably when the H_2_:CO ratios were changed. According to the TEM pictures, the nanoparticles of Pt_3_Ni/K and Pt_3_Ni/G had the edge corner and were most likely rhombus-shaped. As a result of this, the octahedral morphology developed^27,33,46^. The octahedral Pt_3_Ni/K electrocatalyst was formed in the H_2_:CO compositions ranging from 1:3 to 1:24, as illustrated in Fig. 1c,f, whereas the octahedral Pt_3_Ni/G electrocatalyst was produced in H_2_:CO ratios ranging from 1:1 to 1:24, as seen in Fig. 1i–m, respectively. According to the results, the degree of octahedrality looked to be decreasing as the CO composition increased. Meanwhile, the octahedral morphology of the Pt_3_Ni/V was not fully formed, and most of them had a round corner, resulting in a typical spherical shape. As a result, the types of supported carbon have a substantial influence on the shape of Pt_3_Ni metal.

Furthermore, the H_2_:CO ratio is one of the most essential factors in controlling particle shape and size, particularly in solid-state techniques The adsorption of CO on Ni and Pt metals has long been recognized to cause octahedral formation^27^. As shown by the CO adsorption in the Pt-Ni precursor, Ni tends to develop as Ni (111), while Pt prefers to develop as Pt (100)^47,48^. Meanwhile, H_2_ aids in the migration of both metal precursors (Ni and Pt) onto carbon supports and their subsequent reduction into alloys^28,33,46^. In comparison to H_2_/CO flowing gases, the particle size of Pt_3_Ni with flowing pure CO was smaller, and the morphology resembled a blend of octahedron and polyhedron as shown in Fig. 1g,n,u. On the other hand, when pure H_2_ was added during reduction, the particle sizes increased considerably and the morphology seemed to be spherical owing to the absence of CO. Furthermore, the formation of polyhedral or spherical shapes on Vulcan substrate might be caused by a restricted supply of metal precursors, especially Ni, to growth sites, as well as inadequate migration. This indicates that the 3:1 atomic ratio of Pt to Ni is insufficient for the Vulcan carbon-forming octahedron.

**Figure 3:**
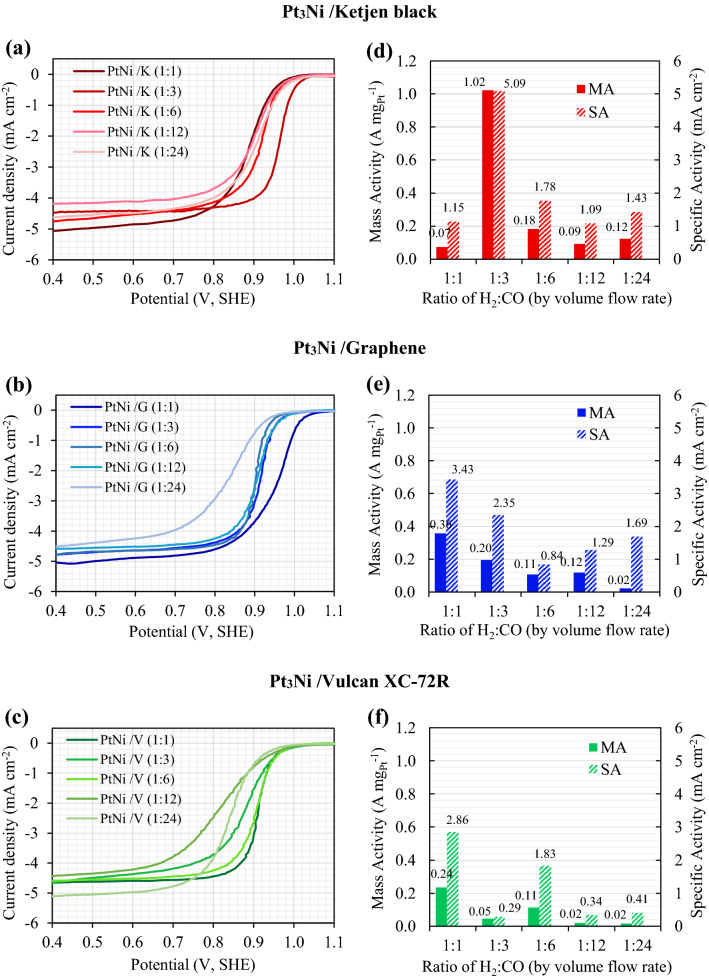
The linear sweep voltammograms (LSV) of Pt_3_Ni on Ketjen black, Graphene and Vulcan XC-72R supports with varying H_2_:CO ratios is shown in (**a**), (**b**), and (**c**); where (**d**), (**e**), and (**f**) show the ORR activities of mass activity (MA) and specific activity (SA).

The linear sweep voltammograms (LSV) were used to determine the electrocatalytic activity of the ORR. After normalization and electrolyte resistance adjustment, the LSV results for Pt_3_Ni/Ketjen black, Pt_3_Ni/Graphene, and Pt_3_Ni/Vulcan XC-72R were plotted as shown in Fig. 3a,b,c. Additionally, the H_2_:CO compositions in the ratios of 1:1, 1:3, 1:6, 1:12, and 1:24 were studied. The free kinetic current ($i_{k}$) for mass transport was computed using Eq. (1)^32,49^.

1 $$\frac{1}{{i_{k} }} = \frac{1}{{i_{{0.9V_{SHE} }} }} - \frac{1}{{i_{L} }}$$

where $i_{k}$ is a mass-transport free kinetics current at 0.9 V_SHE_, $i_{{0.9V_{SHE} }}$ is an experimental current from linear sweep voltammograms at 0.9 V_SHE_, and $i_{L}$ is a diffusion limiting current recorded between 0.2 and 0.7 V_SHE_. As a result, mass activity (MA) and specific activity (SA) were calculated using Eqs. (2) and (3), respectively^50,51^.

2 $$MA = \frac{{i_{k} }}{{m_{Pt} }}$$

where MA is the ORR catalytic mass activity (A mg_Pt_^−1^) of the electrocatalyst and $m_{Pt}$ is the mass loading of Pt on working electrode.

3 $$SA = \frac{MA}{{ECSA}}$$

where SA is the ORR catalytic specifics activity (mA cm^−2^) of the electrocatalyst and ECSA is the electrochemical active surface area of the catalyst measured by hydrogen under potential deposition (HUPD) method.

The mass and specific activity of Pt_3_Ni electrocatalysts on Ketjen black, Graphene, and Vulcan XC-72R are shown in Fig. 3d,e,f, respectively, as a function of the H_2_:CO ratio. It was discovered that Pt_3_Ni electrocatalysts on various carbon supports exhibited distinct maximal ORR activity at various H_2_:CO ratios, with Ketjen black having a 1:3 ratio and Graphene and Vulcan XC-72R having a 1:1 ratio. Clearly, Pt_3_Ni onto Ketjen black (with an H_2_:CO ratio of 1:3) had the highest ORR catalytic activity, with MA of around 1.02 A mg_Pt_^−1^ and SA of approximately 5.09 mA cm^−2^, as shown in Fig. 3d. According to the TEM images in Fig. 1c,i, p, Pt_3_Ni particles were completely dispersed onto carbon supports and clearly revealed an octahedral form for Pt_3_Ni/K (1:3) and Pt_3_Ni/G (1:1). On the other hand, Pt_3_Ni/V (1:1) particles were generally spherical in form and had a particle size as tiny as 3.7 nm. According to C. Zhang et al.^27,28^, the octahedral Pt_3_Ni electrocatalysts should have a particle size of around 6 to 10 nm. As a result, it is once again proven that Pt_3_Ni/V (1:1) was unlikely to have an octahedral structure.

Results show that carbon supports not only affect the physical characteristics of Pt_3_Ni nanoparticles, but also the ORR electrocatalytic activities. The ORR activities of Pt_3_Ni nanoparticles on various carbon supports were optimum at distinct H_2_:CO ratios. For this reason, the H_2_:CO ratios that are optimal for each carbon support to produce the Pt_3_Ni/C electrocatalyst should be taken into consideration. The following section discusses in further detail the comparison of Pt_3_Ni nanoparticles on each carbon support that had the highest ORR catalytic activity.

### Effects of Pt_3_Ni nanoparticles on carbon supports

**Figure 4:**
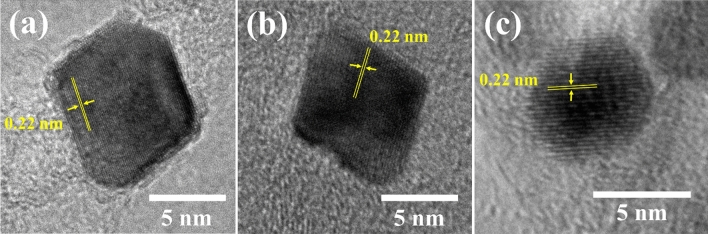
The high-resolution TEM pictures of Pt_3_Ni nanoparticles on each carbon support that demonstrated the highest ORR activity: (**a**) Pt_3_Ni/Ketjen black (1:3), (**b**) Pt_3_Ni/Graphene (1:1) and (**c**) Pt_3_Ni/Vulcan XC-72R (1:1).

**Figure 5:**
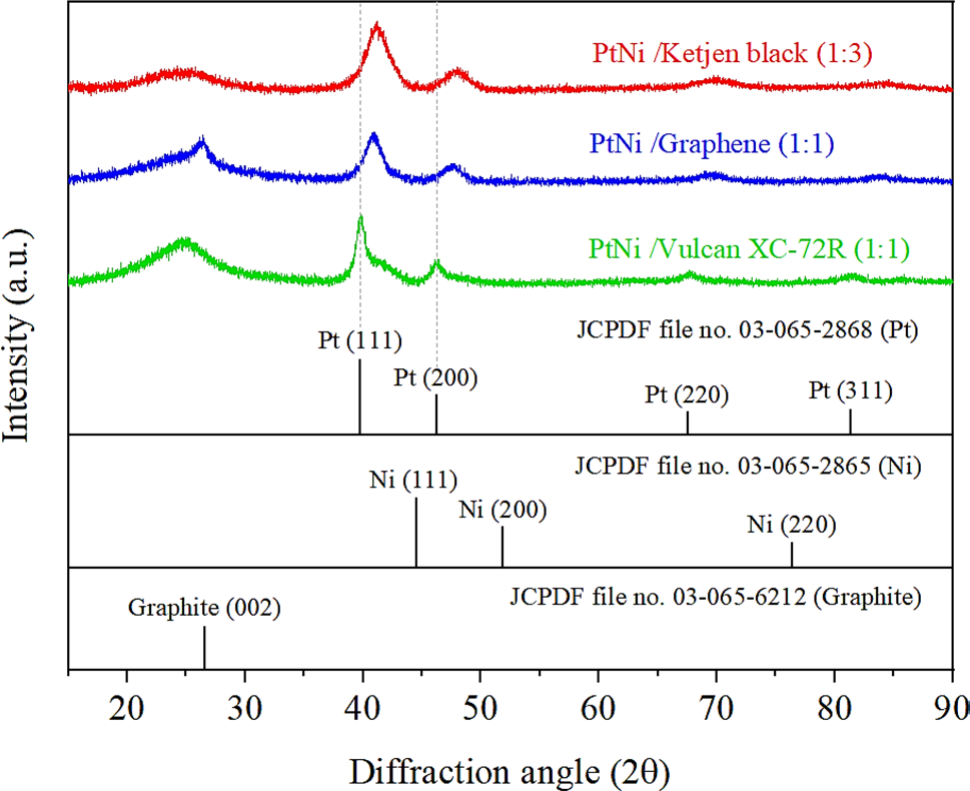
The X-ray diffraction patterns of Pt_3_Ni/C with reference of Pt and Ni crystalline structure from JCPDF: (red line) Pt_3_Ni/Ketjen black (1:3), (blue line) Pt_3_Ni/Graphene (1:1) and (green line) Pt_3_Ni/Vulcan XC-72R.

The three samples with the highest ORR activity (i.e., Pt_3_Ni/K (1:3), Pt_3_Ni/G (1:1), and Pt_3_Ni/V (1:1)) were further determined. The high-resolution TEM pictures on individual particles in Fig. 4 show that the particle morphology of Pt_3_Ni/K (1:3) and Pt_3_Ni/G (1:1) was clearly octahedron, while Pt_3_Ni/V (1:1) was spherical. These three photos showed a lattice spacing of 0.22 nm, indicating the surface of Pt-Ni nanoparticles with (111) facets ^10,27,28,31,32^. Furthermore, XRD analysis of the powders of those catalysts revealed that they had a face-centered cubic (fcc) structure, as shown in Fig. 5. The shift of the Pt (111) diffraction peak of Pt_3_Ni/K (1:3) and Pt_3_Ni/G (1:1) to the Ni (111) peak indicates the creation of a Pt-Ni alloy. On the other hand, the Pt (111) diffraction peak of Pt_3_Ni/V (1:1) did not shift to Ni (111), but the spectrum seemed to split into two peaks, one for Pt (111) and another for Ni (111). This indicates that Pt and Ni were only weakly bound and did not form the Pt-Ni alloy but rather formed a bimetallic structure.

**Figure 6:**
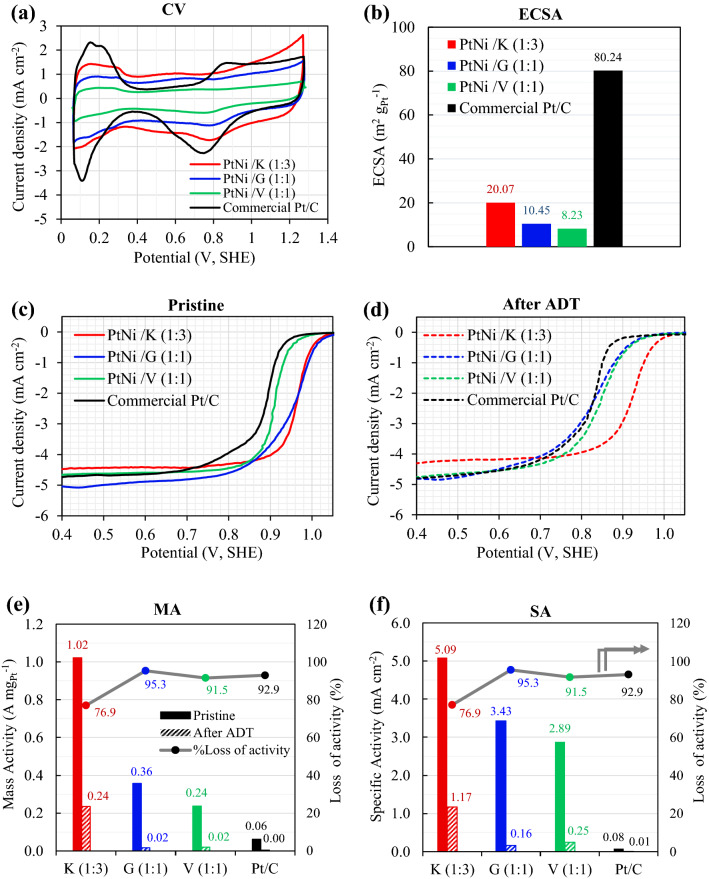
The voltammograms and electrochemical activities of Pt_3_Ni/C compared to commercial catalyst of 20 wt%. Pt/C: (**a**) cyclic voltammograms, (**b**) the ECSA, (**c**) and (**d**) linear sweep voltammograms of pristine and ADT samples, respectively, (**e**) mass activity (MA) and (**f**) specific activity (SA).

As shown in Fig. 6, the electrocatalytic properties of Pt_3_Ni/K (1:3), Pt_3_Ni/G (1:1), and Pt_3_Ni/V (1:1) were compared to those of a commercial Pt/C catalyst. The cyclic voltammograms (CV), Fig. 6a, were scanned at a rate of 50 mV s^−1^, and the electrochemical active surface area (ECSA) was derived from the hydrogen under potential deposition (HUPD) using the following equation^6,27,50^.

4 $$ECSA = \frac{{Q_{H} }}{{m_{Pt} \times 210}}$$

The ECSA (m^2^ g_Pt_^−1^) is the electrochemical active surface area of electrocatalysts, $Q_{H}$ (Coulomb) is the charge of integrated hydrogen desorption area, $m_{Pt}$ (grams) is the mass loading of Pt in GC-RDE working electrode, and 210 (µC cm^−2^) is a charge density conversion factor during hydrogen adsorption–desorption area of Pt polycrystalline.

Figure 6a, all Pt_3_Ni catalysts underwent hydrogen adsorption–desorption at potentials ranging from 0.05 to 0.4 V_SHE_. In Fig. 6b, the commercial Pt/C catalyst had the greatest ECSA at roughly 80.24 m^2^ g_Pt_^−1^, followed by Pt_3_Ni/K (20.07 m^2^ g_Pt_^−1^), Pt_3_Ni/G (10.45 m^2^ g_Pt_^−1^), and Pt_3_Ni/V (8.23 m^2^ g_Pt_^−1^). It was also found that the Pt_3_Ni/K had the largest double layer, followed by Pt_3_Ni/G, commercial Pt/C, and Pt_3_Ni/V, respectively. The double layer was different because of the different structures of the carbon supports that were used. This was seen in a double layer comparison between Pt_3_Ni/V and commercial Pt/C, where the carbon support was the same Vulcan XC-72R. The likelihood for double layer formation was shown to be connected to the specific surface area of carbon supports, with Ketjen black having the greatest specific surface area (1499.11 m^2^ g^−1^) and the biggest double layer.

The ORR electrocatalytic activity of the pristine samples (after 40 cycles of activation) was investigated using the LSV, as shown in Fig. 6c. After 4000 voltage cycles, the accelerated durability test (ADT) was performed, and the LSV of ADT sample was evaluated as shown in Fig. 6d. The mass activity (MA) and specific activity (SA) of pristine and ADT samples were computed using Eqs. (2) and (3), and the results are presented in Fig. 6e,f, respectively. In the pristine sample, it was discovered that the Pt_3_Ni/K (1:3) catalyst had the greatest MA of 1.02 A mg_Pt_^−1^, followed by the Pt_3_Ni/G (1:1) and the Pt_3_Ni/V (1:1) catalysts, and that the commercial Pt/C catalysts had the lowest MA of 0.062 A mg_Pt_^−1^, as shown in Fig. 6e. Furthermore, the SA in Fig. 6f, which represents the normalization of MA by ECSA, showed the same trend as the MA. In terms of SA, the pristine results reveal that Pt_3_Ni/K (1:3) yielded the greatest value of 5.09 mA cm^−2^, which was 1.5 times higher than Pt_3_Ni/G (1:1), 1.8 times higher than Pt_3_Ni/V, and 66.1 times higher than commercial Pt/C catalyst.

The results indicate that all Pt_3_Ni/C samples had much higher ORR electrocatalytic activity for MA and SA than commercial Pt/C catalysts, despite the commercial catalysts had the greatest ECSA. This is most likely owing to the fact that the electronic structure of Pt is altered when Ni is added, resulting in the formation of a Pt-Ni alloy or bi-metal. This enhances oxygen molecule binding onto the metal surface^38,42^. Additionally, the formation of an octahedral shape, such as the (111) plane in a Pt-Ni alloy, had a significant effect on the ORR activity^10,27–38,42,52^. Thus, Pt_3_Ni/K (1:3) and Pt_3_Ni/G (1:1) demonstrated much higher ORR activity (both MA and SA) than Pt_3_Ni/V (1:1), which had a spherical form and was not an alloy catalyst (bi-metal).

SA and MA were computed after 4,000 cycles and are displayed in the right-hand histograms of Fig. 6e,f, respectively. Pt_3_Ni/K (1:3) retained the greatest MA and SA values of 0.24 A mg_Pt_^−1^ and 1.17 mA cm^−2^, respectively. On the other hand, commercial Pt/C catalysts produced the least MA (0.004 A mg_Pt_^−1^) and SA (0.005 mA cm^−2^). According to the results between pristine and after ATD, the ORR activity losses were evaluated and plotted on the right-hand side scale of Fig. 6e,f. Pt_3_Ni/K (1:3) loses around 76.9 percent of its activity, whereas Pt_3_Ni/G (1:1) and Pt_3_Ni/V (1:1) lose approximately 95.3 percent and 91.5 percent of their activity, respectively. This indicates that Pt_3_Ni/K (1:3) had the greatest stability and durability, followed by Pt_3_Ni/V (1:1) and Pt_3_Ni/G (1:1).

**Figure 7:**
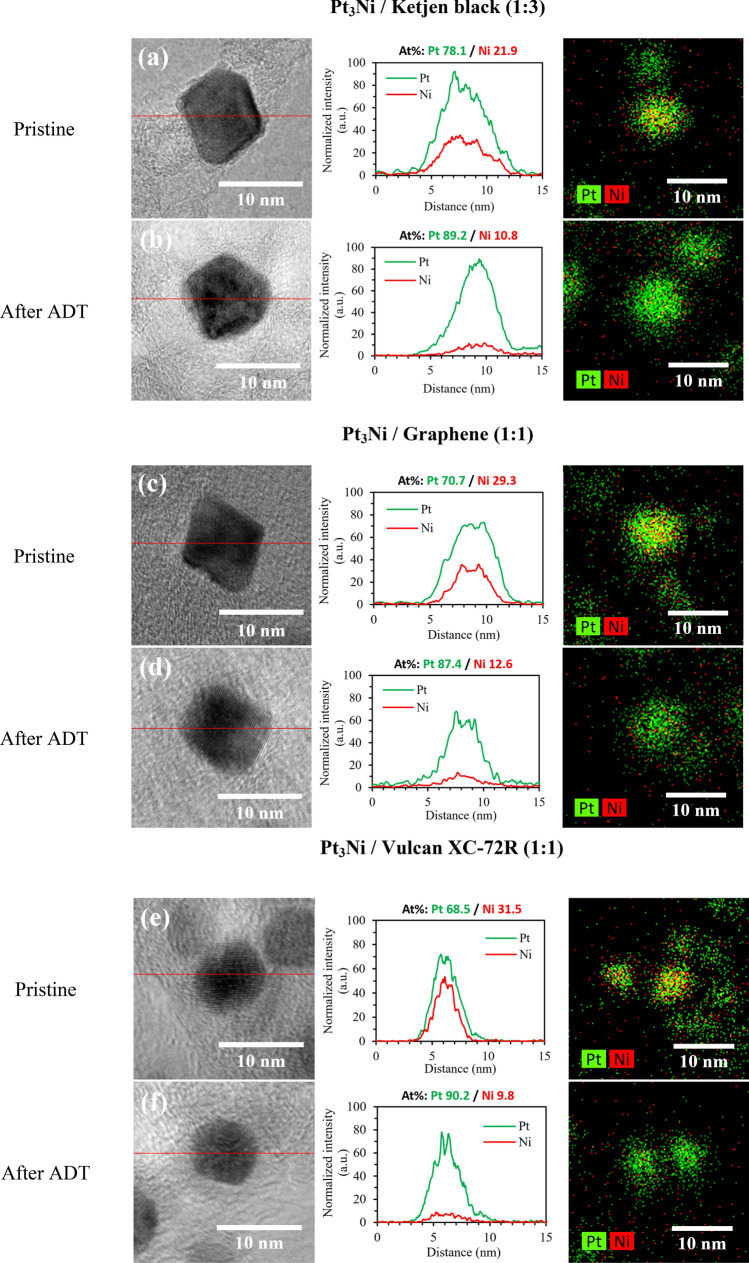
The TEM images, line scanning profile with EDX analysis, and spectroscopic mapping of Pt_3_Ni /C nanoparticles between pristine and after ADT samples; (**a**, **b**) Pt_3_Ni/Ketjen black (1:3); (**c**, **d**) Pt_3_Ni/Graphene (1:1); and (**e**, **f**) Pt_3_Ni/Vulcan XC-72R (1:1).

The elemental mapping images and line scanning profiles in Fig. 7 show that Pt and Ni in pristine samples were uniformly distributed across the carbon surface, and Pt surface covered over Ni as a core shell catalyst. A Pt-rich surface layer was detected after ADT, with Ni atoms dissolving in the interior region. As a result, the favored octahedral structure collapsed, and shape-controlled catalysts began to acquire a spherical shape. Therefore, the octahedral nanoparticle's surface energy was lowered^53,54^ and the ORR activity was deteriorated as a result. In Fig. 7a,b, Pt_3_Ni/K (1:3) seemed to have a less collapsed octahedral form than Pt_3_Ni/G. (1:1). Furthermore, the Ni leached away the most in the Pt_3_Ni/V (1:1) sample, resulting in lost bimetal behavior and dramatically reduced ORR activity. As a result, it verifies once again that Pt_3_Ni/K (1:3) exhibited the greatest stability and durability of these samples.

## Conclusion

The gas phase synthesis was shown to be an effective method for producing an octahedral Pt_3_Ni/C electrocatalyst for ORR at the cathode side of PEM fuel cells. Regardless of H_2_:CO composition, the Pt_3_Ni on Ketjen black had the highest particle size, followed by Graphene and Vulcan XC-72R, which corresponded to specific surface area. On Ketjen black and Graphene, octahedral Pt_3_Ni nanoparticles with alloy behavior were clearly detected, whereas spherical particles with bimetallic behavior were identified on Vulcan XC-72R. The ORR activities of Pt_3_Ni nanoparticles on each carbon support were best at distinct H_2_:CO ratios, which were 1:3 for Pt_3_Ni/K and 1:1 for Pt_3_Ni/G and Pt_3_Ni/V. The ORR activity of Pt_3_Ni/K (1:3) was the greatest, followed by Pt_3_Ni/G (1:1) and Pt_3_Ni/V (1:1). These Pt_3_Ni /C, on the other hand, lose their ORR activity after ADT voltage cycles. Their stability is harmed by the collapse of the octahedral structure. As a result, additional efforts to improve stability will be required. Doping or infusing the third or fourth metals is likely to prevent the octahedral structure from collapsing.

## Supplementary Information

Supplementary Information.

## Data Availability

The datasets generated and analyzed during the current study are available in the Zenodo repository, https://zenodo.org/record/6462897#.Ylks1ehBzb0. https://doi.org/10.5281/zenodo.6462897 (Supplementary information).
