# Supplementary material for: The effect of supporting carbons on the gas phase synthesis of octahedral Pt3Ni electrocatalysts with various H2:CO ratios
Source: Sci Rep. 2022 Jul 22;12:12504. doi: 10.1038/s41598-022-16742-x (PMC9307833; doi:10.1038/s41598-022-16742-x)
Supplement: Supplementary file 1 — Supplementary Information. [file 41598_2022_16742_MOESM1_ESM.docx]

**Supporting Information for**

**The effect of supporting carbons on the gas phase synthesis of octahedral Pt_3_Ni electrocatalysts with various H_2_:CO ratios**

L. Payattikul ^1,2,3^, L. Intakhuen^1,3^, T. Kiatsiriroat ^1,4^, K. Punyawudho ^1,3, *^

*^1^ Department of Mechanical Engineering, Faculty of Engineering, Chiang Mai University, 50200, Thailand*

*^2^ Graduated School, Chiang Mai University, 50200, Thailand*

*^3^ Energy Harvesting and Storage Laboratory, Mechanical Engineering, Chiang Mai University, 50200, Thailand*

*^4^ Center of Clean Energy Development for Green, Faculty of Engineering, Chiang Mai University, Chiang Mai, 50200, Thailand*


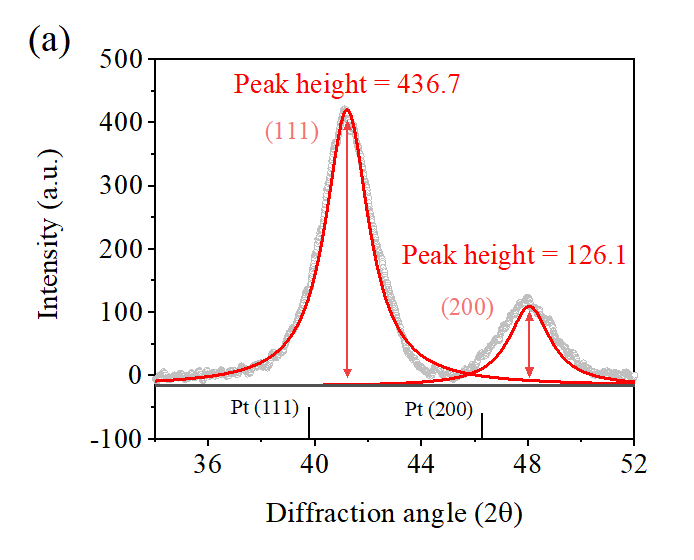

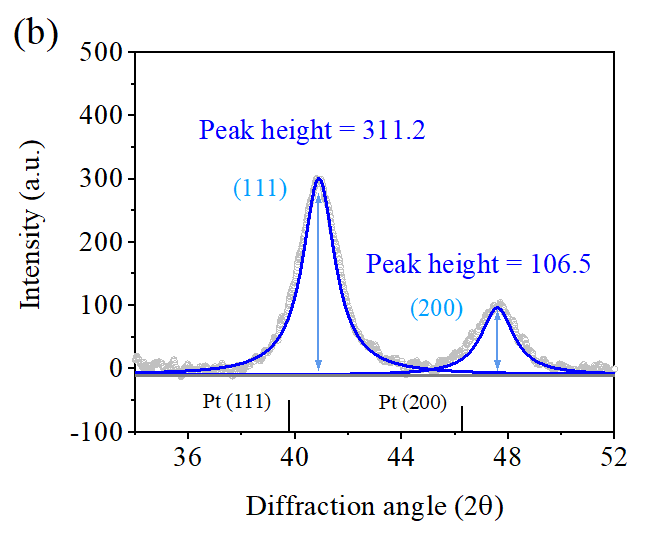


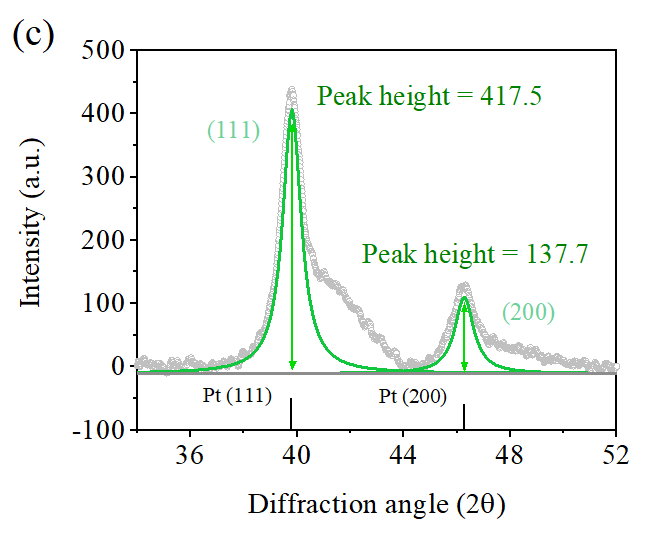


**Figure S1.** The diffraction peak Pt(111) and Pt(200) from XRD patterns of prepared Pt_3_Ni/C catalyst: (a) Pt_3_Ni /Ketjen black, (b) Pt_3_Ni /Graphene and (c) Pt_3_Ni /Vulcan XC-72R

**Figure S2.** (a) The cyclic voltammograms and (b) the ECSA of Pt_3_Ni/C compared to commercial catalyst of 20%wt. Pt/C after 4000 cycles of ADT

**Figure S3.** Comparison of the ORR activity both MA and SA of Pt-Ni electrocatalysts

**Figure S4.** Comparison of the ORR activity both MA and SA of Commercial Pt/C

|  | | | |
| --- | --- | --- | --- |
| **Lot number of preparation** | **1^st^** | **2^nd^** | **3^rd^** |
| Pt_3_Ni /Ketjen black | 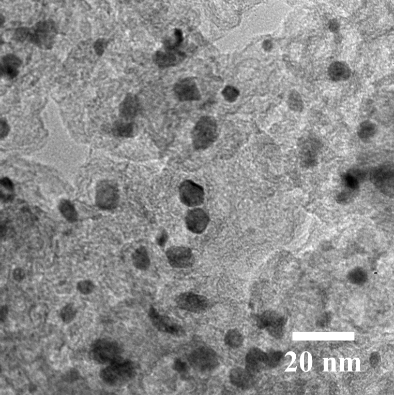 | 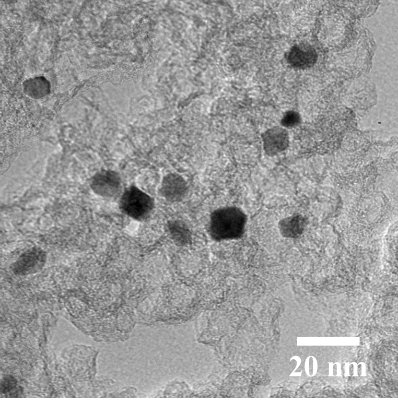 | 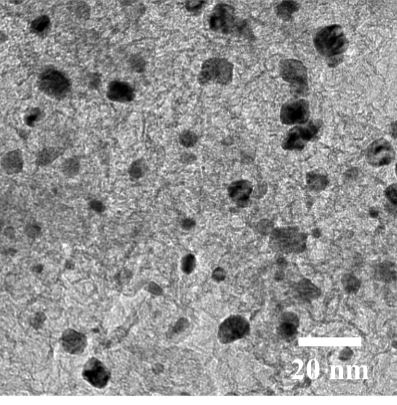 |
| Pt_3_Ni /Graphene | 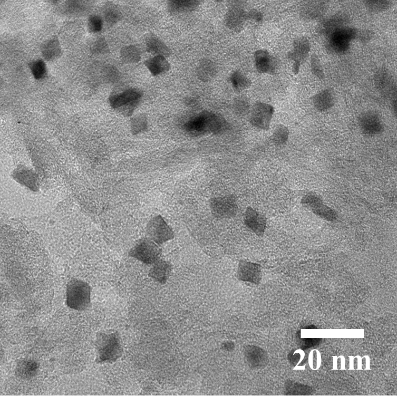 | 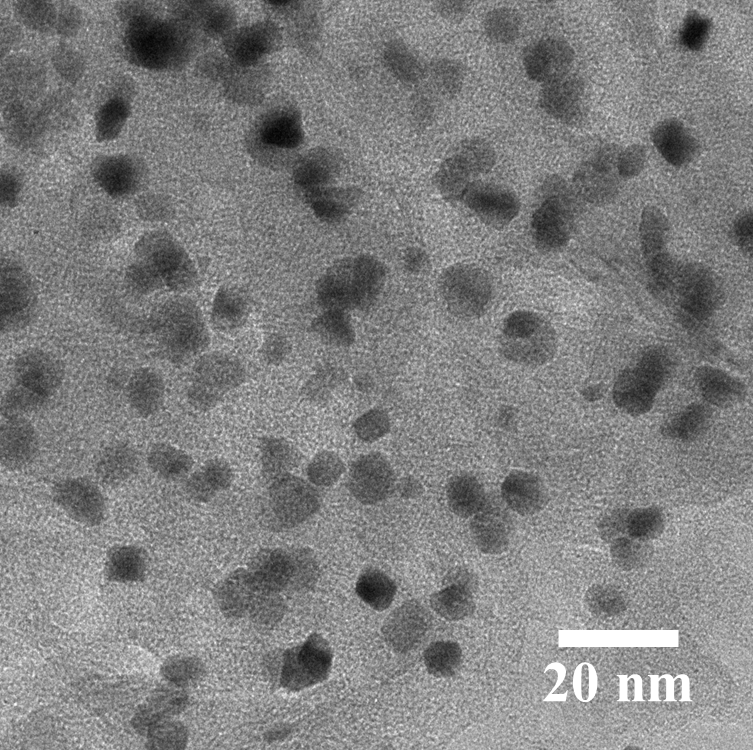 | 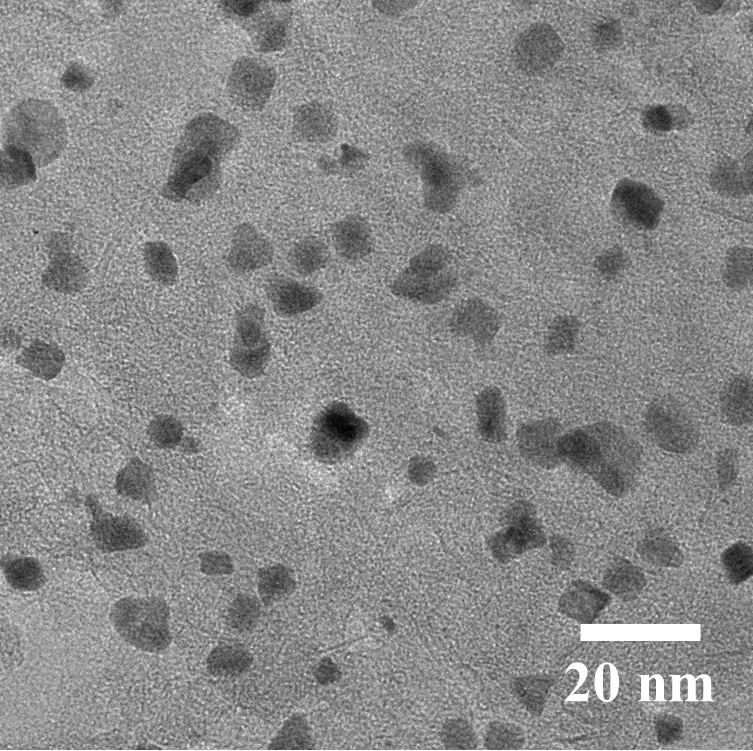 |
| Pt_3_Ni /Vulcan XC-72R | 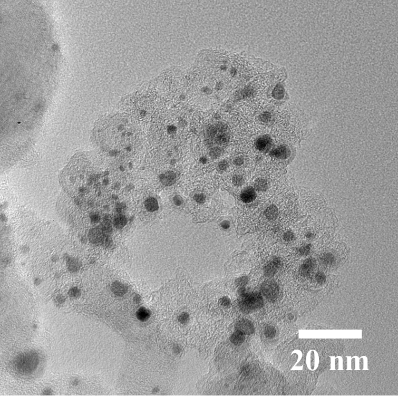 | 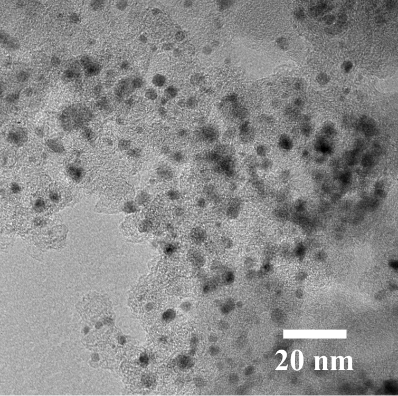 | 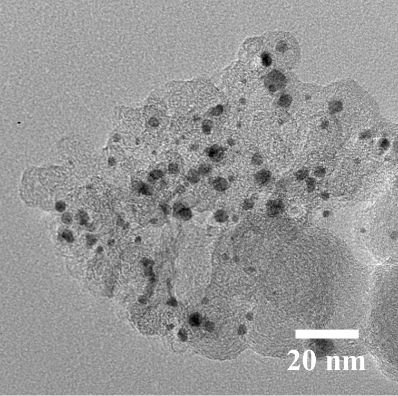 |

**Figure S5.** The TEM images of three batches of Pt_3_Ni/C catalysts, each with the magnification of 200,000 times.

**Table S1.** The peak position, peak intensity and Pt(111) to Pt(200) ratio from XRD patterns of prepared Pt_3_Ni/C catalysts.

| Samples | Peak position | | The shift of peak position from reference peak* | | Peak intensity | | Intensity ratio of Pt(111) to Pt(200) |
| --- | --- | --- | --- | --- | --- | --- | --- |
|  | (111) | (200) | (111) | (200) | (111) | (200) |  |
| Pt_3_Ni /K | 41.20° | 48.05° | +1.41° | +1.78° | 436.7 | 126.1 | 3.46 |
| Pt_3_Ni /G | 40.89° | 47.59° | +1.10° | +1.31° | 311.2 | 106.5 | 2.92 |
| Pt_3_Ni /V | 39.80° | 46.27° | +0.01° | 0 | 417.5 | 137.7 | 3.03 |

* Reference peak position of Pt (111) and Pt (200) are 39.79° and 46.27°, respectively

**Table S2.** Comparison of structure and ORR activity of PtNi electrocatalysts

| Structure of Nanoparticles | | ORR Activity | | LSV Test conditions | | Ref. |
| --- | --- | --- | --- | --- | --- | --- |
| Shape | Average size (nm) | MA  (A mg_Pt_^-1^) | SA  (mA cm^-2^) | Electrolyte solution | Scan rate  (mV s^-1^) |  |
| **Octahedral** | **9.9** | **1.02** | **5.09** | **0.1 M HClO_4_** | **20** | **This work** |
| Tetrahedron &  Octahedron | 8.5 | 1.22 | 5.01 | 0.1 M HClO_4_ | 10 | [14] |
| Rhombic dodecahedral | 17.9 | 0.91 | 1.39 | 0.1 M HClO_4_ | 10 | [24] |
| Nanowires | 2~3 (diameter) | 0.33 | 0.60 | 0.1 M HClO_4_ | 5 | [25] |
| Octahedral | 6.0 | 0.46 | 0.95 | 0.1 M HClO_4_ | 10 | [32] |
| Octahedral | 7.1 | 0.92 | 2.42 | 0.1 M HClO_4_ | 10 | [33] |
| Octahedral | 7.0 | 0.41 | 1.07 | 0.1 M HClO_4_ | 5 | [34] |
| Octahedral | 12.8 | 0.48 | 1.38 | 0.1 M HClO_4_ | 5 | [35] |
| Octahedral | 10-15 | 1.68 | 5.84 | 0.1 M HClO_4_ | 5 | [36] |
| Octahedral | 6.4 | 0.52 | 1.07 | 0.1 M HClO_4_ | 5 | [37] |
| Cub-octahedral | 5.5 | 1.16 | 0.84 | 0.1 M HClO_4_ | 20 | [38] |
| Octahedral | 6.0 | 0.64 | 1.11 | 0.1 M HClO_4_ | 20 | [41] |

**Table S3.** Comparison ORR activity of commercial Pt/C catalysts

| ORR Activity | | LSV Test conditions | | Ref. |
| --- | --- | --- | --- | --- |
| MA  (A mg_Pt_^-1^) | SA  (mA cm^-2^) | Electrolyte solution | Scan rate  (mV s^-1^) |  |
| **0.062** | **0.077** | **0.1 M HClO_4_** | **20** | **This work** |
| 0.062 | 0.105 | 0.5 M H_2_SO_4_ | 20 | [6] |
| 0.103 | 0.177 | 0.1 M HClO_4_ | 10 | [14] |
| 0.180 | 0.380 | 0.1 M HClO_4_ | 20 | [21] |
| 0.180 | 0.340 | 0.1 M HClO_4_ | 10 | [22] |
| 0.037 | 0.052 | 0.1 M HClO_4_ | 10 | [24] |
| 0.140 | 0.270 | 0.1 M HClO_4_ | 5 | [25] |
| 0.106 | 0.146 | 0.1 M HClO_4_ | 10 | [32] |
| 0.086 | 0.162 | 0.1 M HClO_4_ | 5 | [34] |
| 0.085 | 0.209 | 0.1 M HClO_4_ | 5 | [36] |
| 0.086 | 0.162 | 0.1 M HClO_4_ | 5 | [37] |
| 0.172 | 0.197 | 0.1 M HClO_4_ | 20 | [41] |
